# Tissue culture coupled with a gas exchange system offers new perspectives on phenotyping the developmental biology of *Solanum lycopersicum* L. cv. ‘MicroTom’

**DOI:** 10.3389/fpls.2022.1025477

**Published:** 2022-11-10

**Authors:** Marco Pepe, Telesphore R. J. G. Marie, Evangelos D. Leonardos, Mohsen Hesami, Naheed Rana, Andrew Maxwell Phineas Jones, Bernard Grodzinski

**Affiliations:** Department of Plant Agriculture, University of Guelph, Guelph, ON, Canada

**Keywords:** tomato, MicroTom, tissue culture, gas exchange, fruit production, metabolites, photosynthesis, respiration

## Abstract

*Solanum lycopersicum* L. cv. ‘Microtom’ (MicroTom) is a model organism with a relatively rapid life cycle, and wide library of genetic mutants available to study different aspects of plant development. Despite its small stature, conventional MicroTom research often requires expensive growth cabinets and/or expansive greenhouse space, limiting the number of experimental and control replications needed for experiments, and can render plants susceptible to pests and disease. Thus, alternative experimental approaches must be devised to reduce the footprint of experimental units and limit the occurrence problematic confounding variables. Here, tissue culture is presented as a powerful option for MicroTom research that can quell the complications associated with conventional MicroTom research methods. A previously established, non-invasive, analytical tissue culture system is used to compare *in vitro* and conventionally produced MicroTom by assessing photosynthesis, respiration, diurnal carbon gain, and fruit pigments. To our knowledge, this is the first publication that measures *in vitro* MicroTom fruit pigments and compares diurnal photosynthetic/respiration responses to abiotic factors between *in vitro* and *ex vitro* MicroTom. Comparable trends would validate tissue culture as a new benchmark method in MicroTom research, as it is like Arabidopsis, allowing replicable, statistically valid, high throughput genotyping and selective phenotyping experiments. Combining the model plant MicroTom with advanced tissue culture methods makes it possible to study bonsai-style MicroTom responses to light, temperature, and atmospheric stimuli in the absence of confounding abiotic stress factors that would otherwise be unachievable using conventional methods.

## Introduction

*Solanum lycopersicum* L. cv. ‘Microtom’ (MicroTom) is a miniature, herbaceous tomato cultivar, with a relatively rapid life cycle from seed germination to seed set. Like *Arabidopsis*, MicroTom is a model organism with a well annotated genome and extensive mutant seedbank for studying plant-pathogen interactions (Takahashi et al., 2005) as well as genetic (Dan et al., 2006), molecular (Park et al., 2007), and physiological (Vitale et al., 2022) aspects of vegetative growth, flowering, and fruiting stages of the plant’s life cycle. Despite the miniature growth form of this cultivar, MicroTom experiments often rely on conventional cultivation methods that require expansive bench space and/or expensive growth cabinets to create replicated environments. These requirements limit the number of practically achievable experimental units, compromising experimental design and statistical analysis. Additionally, using conventional controlled systems runs the risk of exposing plants to undesired pests and disease (Arie et al., 2007) that can jeopardize successful rearing and study of sensitive mutants, selected specimens, and bioengineered lines. These potential limitations necessitate alternative investigational approaches that reduce the footprint of experimental units needed to obtain reliable data and curb the occurrence confounding variables.

Tissue culture offers an ideal option to manage the complications associated with traditional MicroTom research techniques. This approach allows plants to be produced in a highly controlled axenic environment, in the absence of confounding biotic and abiotic stress factors, while maintaining specific light, temperature and atmospheric stimuli to study complex plant dynamics with a high level of control and replicability (Monthony et al., 2021; Pepe et al., 2021; Pepe et al., 2022). By combining the model MicroTom with tissue culture gas exchange techniques, it is possible to comprehensively study bonsai-style MicroTom responses to light, temperature, and atmospheric stimuli at every developmental stage. Comparing photosynthetic and respiratory responses and fruit pigment profiles of tissue culture and conventionally grown MicroTom is a necessary preliminary step in devising dynamic research methods to overcome the associated setbacks of traditional systems. Similar trends observed between *in vitro* and *ex vitro* MicroTom would validate tissue culture as a new benchmark method in MicroTom research for narrowing down multifactorial treatment combinations before scaling up to commercial experiments.

Here, a previously established, non-invasive, analytical tissue culture system (Pepe et al., 2022) was used to compare *in vitro* and cabinet -produced MicroTom, both grown under their respective normal conditions and practices, by assessing photosynthesis and respiration at two different light intensities (acclimated and doubled) for a diurnal 48-hr period. Additionally, tissue culture produced MicroTom fruit pigments are measured for the first time. Ultimately, the preliminary data presented involving MicroTom fruit pigments and comparing *in vitro* and *ex vitro* MicroTom metabolism paves the way for an alternate system to easily assess plant development, from seed germination to fruit production.

## Whole plant(let) gas exchange is a powerful phenotyping platform

**Figure 1:**
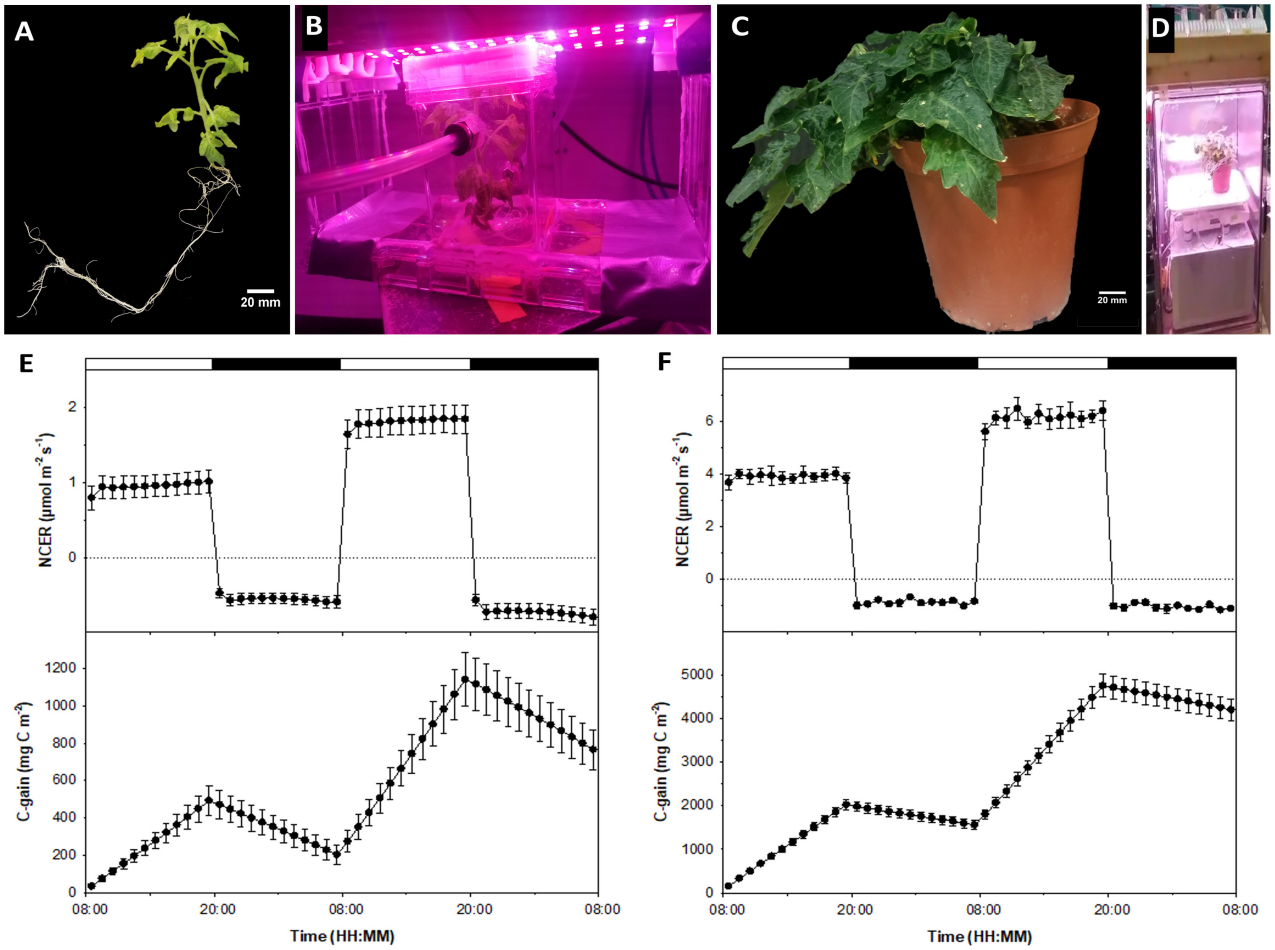
Comparing tissue culture and conventional whole-plant systems. Shown are **(A)** tissue culture MicroTom grown from seed; **(B)** *in vitro* MicroTom connected to the non-invasive tissue culture gas exchange system using the methods presented by Pepe et al. (2022) and Leonardos and Grodzinski (2014); **(C)** 8-week old MicroTom grown with potting soil in growth cabinets; **(D)** potted MicroTom in whole-plant gas-exchange system (see Leonardos and Grodzinski, 2016) equipped with white-red LED fixtures (Lighting Science Group Company, RI, USA); **(E)** tissue culture NCER and carbon gain; and **(F)** cabinet grown plant NCER and carbon gain. Error bars represent means and standard errors of four replicates.

The majority of plant dry weight is comprised of carbon, hydrogen, and oxygen, much of which is assimilated from the atmosphere. By assessing CO_2_ exchange rates throughout daytime photosynthetic and nighttime respiratory periods, growth dynamics of biological plant systems can be assessed (Dutton et al., 1988; Leonardos and Grodzinski, 2016). These techniques can also be used to evaluate *in vitro* plants, since the tissue culture micro-environment confers significant influence over plantlet growth and development (Walli et al., 2019). Comparing gas exchange between tissue culture plantlets (**Figures 1A, B**) and cabinet grown plants (**Figures 1C, D**) in response to experimental conditions allows for modeling similar photosynthesis and respiration trends, validating the *in vitro* MicroTom system as a parallel or alternative experimental platform.

Gas exchange data shows similar trends between *in vitro* and *ex vitro* specimens, with net carbon exchange rates (NCER) remaining steady throughout differential light and dark periods. Positive NCER, representing net photosynthesis (Pn), increased in response to higher light intensities and showed a higher negative NCER, representing dark respiration (Rd), during dark periods in response to the previous photoperiod’s light intensity increase (**Figures 1E, F**).

## Similar trends in photosynthetic responses to a doubling of light intensity

Pn of growth cabinet plants were 4.04 times higher than tissue culture counterparts at their respective acclimated light intensities of 200 µmol m^-2^ s^-1^ and 50 µmol m^-2^ s^-1^ photosynthetic photon flux density (PPFD), which also differ by a factor of 4 (**Figures 1E, F**). When light intensities were doubled to 100 µmol m^-2^ s^-1^ *in vitro* and 400 µmol m^-2^ s^-1^ *ex vitro*, Pn increase was 3.54 times different between counterpart plants (**Figures 1E, F**). Cultured plant Pn increased by a factor of roughly 1.91 (**Figure 1E**), while growth cabinet plant Pn increased roughly by a factor of 1.58 (**Figure 1F**). These are similar trends, and the discrepancies may be attributed to different physiological differences such as differing light intensity acclimation, which can be elucidated with future light curves.

Throughout their development, growth cabinet plants would have been exposed to [CO_2_] in a similar range to that tested during experimentation, whereas [CO_2_] in “sealed” tissue culture vessels regularly fluctuate and are largely dictated by CO_2_ evolution during darkness and CO_2_ assimilation during light periods (Morini and Melai, 2005), leading to low [CO_2_] during the photoperiod. This would perhaps allow the experimental [CO_2_] of 400 ppm to act as augmented [CO_2_] for *in vitro* plants relative to what they would normally experience. Thus, this experiment should be repeated with in ventilated culture vessels or using forced air CO_2_ supplementation.

Additionally, the slight relative difference in Pn could be due to differences in mutual canopy shading, which can reduce the amount of light available to leaves in different layers of the canopy (Song et al., 2016). Larger and more developed canopies of *ex vitro* plants compared to *in vitro* plantlets (**Figures 1A, C**) may have restricted leaf interception and absorbance of light, decreasing optimal use of available irradiance. Although the aforementioned factors may have contributed to moderate differences in relative Pn between tissue culture and cabinet grown MicroTom, the response patterns were similar overall. Pn of both treatments still increased at similar rates when irradiance was doubled, with rates remaining steady throughout each light treatment. These Pn responses indicate the power of the tissue culture MicroTom system to model light influence that would remain relevant for conventional production practices.

## Similar trends in dark respiration with notable differences

Rd of both cabinet grown plants (**Figure 1F**) and tissue culture plantlets (**Figure 1E**) were higher during the second dark period following higher intensity light treatments compared to the first dark period at the acclimated light intensity. This is indicated by differences in Rd (**Figures 1E, F**), though difficult to see with the cabinet grown plants due to scale bar values that span positive and negative NCER for the whole 2 day period (**Figure 1F**). Additionally, tissue culture plantlets showed an increase in Rd toward the end of the second dark period **(Figure 1E**) and had proportionally higher Rd than those of cabinet grown plants (**Figures 1E, F**). The Rd of the tissue culture plants were about 59% of the Pn on day 1 and about 40% on day 2. In comparison, the Rd of the growth cabinet plants were 23% and 17% of Pn on day 1 and 2, respectively. These observations are mainly due to differences in light intensities of the two treatments, which delivered different Pn rates, but also can impact subsequent Rd activity. The Rd rates of the cabinet plants were higher than those of the tissue culture plants as shown in **Figures 1E, F**. However, rather than expressing Rd on a leaf area basis, a more appropriate way to express Rd and to represent sink activity is to analyze the data on a dry weigh basis, which showed that tissue culture plants had approximately 1.5 times higher Rd than cabinet plants on either night (data not shown).

## The question of media sucrose effects on photosynthesis and respiration

Another area that needs further research is the discernment of supplemental sucrose effects on Pn and Rd. It is standard practice to add sucrose to the tissue culture media (Gago et al., 2014). While sucrose is important to maintain cultured plantlet growth (Rocha et al., 2013), it has been reported to obstruct photosynthesis (Rybczyński et al., 2007). However, previous research has shown that CO_2_ is still a limiting factor if sucrose is in the media under increasing light intensity, meaning that sucrose does not inhibit additional CO_2_ uptake or it is minimal if it does (Pepe et al., 2022). What’s different in the present study, is the diurnal measurement of Pn and subsequent Rd and their responses to a light intensity increase. Accumulation of photosynthates in leaves occurs when photosynthesis exceed sink capacity (Norikane et al., 2010) which was likely the case in both of the *in vitro* and *ex vitro* plants. This can be amplified *in vitro* by the presence of exogenous sucrose (Lembrechts et al., 2017). Media sucrose directly impacts plant tissue carbohydrate accumulation, which can result in augmented Rd throughout dark periods (Kozai et al., 2005). Leaf carbohydrate sequestration from media sucrose, along with the increased Pn of the previous high irradiance period, may have mutually contributed to the increasing Rd values of the *in vitro* plantlets especially toward the end of the second dark cycle (**Figure 1E**). Another interesting hypothesis is a possible circadian influenced increase in respiration in the few hours preceding dawn, that may discriminate between media derived sucrose and photosynthetic derived sucrose. This hypothesis is suggested as there seems to be pre-dawn NCER patterns when plants are grown under robust circadian entraining LED recipes (Marie et al., 2022). Although there are differences in sink activity and the presence of tissue culture media sucrose might have contributed to moderate proportional differences in Rd, informative trends are still observed among tissue cultured and cabinet -grown specimens that open new research directions.

## Carbon gain and loss patterns for non-destructive biomass accumulation

Carbon gain and losses reflect diurnal photosynthesis and respiration in plants (Jiao et al., 1991), which is evident in the data presented (**Figures 1E, F**), demonstrating the ability of either system to measure biomass accumulation non-destructively. In accordance with light intensity and Pn differing by a factor of 4.05 during the first illuminated period (**Figures 1E, F**), carbon gain during this period also differed by a factor of 4.10 between counterpart plants (**Figures 1E, F**). The relative differential increase of carbon gain was approximately 3.40 times during the high irradiance cycle (second light period between tissue culture and cabinet plants) (**Figures 1E, F**). At the end of the experiment, total carbon gain was 5.49 times higher in cabinet grown plants than in tissue culture plants (**Figures 1E, F**), which is a another reflection of the lower Pn and higher relative Rd of the tissue culture plants. Carbon gain trends almost directly reflect NCER trends, as they should, indicating that both tissue culture and whole plant gas exchange systems are functioning properly. This also shows that the tissue culture gas exchange system developed in our laboratory is a highly accurate approach capable of quantifying NCER and carbon gain of cultured plantlets.

## MicroTom fruit pigments are normal in tissue culture

**Figure 2:**
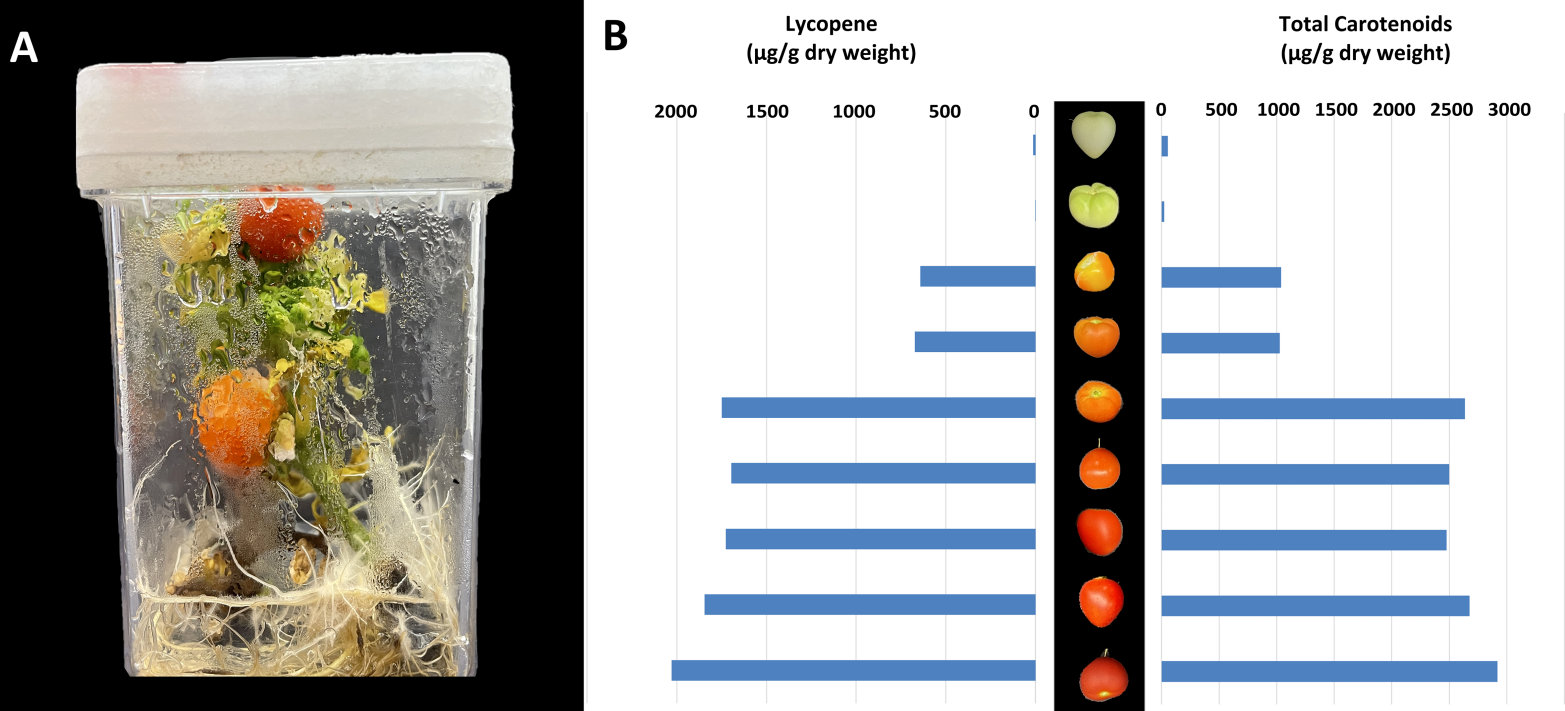
Production and analysis of *in vitro* MicroTom fruit. Indicated are **(A)** example of *in vitro* MicroTom fruit production system, and **(B)** tissue culture produced MicroTom metabolite profiles.

A significant area of focus in the field of tomato production is yield and fruit quality in response to light (Vitale et al., 2022). Since major categories of secondary plant metabolites are present in tomato fruit (Li et al., 2020), the rapid cycling MicroTom is an ideal candidate to study fruit quality in response to different abiotic factors. Although light quality can significantly impact the nutritional value of growth chamber produced MicroTom fruit by increasing antioxidant levels (Vitale et al., 2022), it is unknown if the secondary metabolite activities of *in vitro* MicroTom fruit mimic their *ex vitro* counterparts. This represents an important area of focus for fruit production and quality in response to light, and a research area perfectly suited for the tissue culture approach. Accounts of tissue culture fruiting are relatively rare (Bodhipadma and Leung, 2003), but have been reported in several species. Fruit production *in vitro* has only been evaluated in a limited selection of plants such as *Capsicum* sp. (Tisserat and Galletta, 1995; Bodhipadma and Leung, 2003), *Pisum sativum* L. (Franklin et al., 2000), and non-MicroTom tomatoes (Sheeja and Mandal, 2003; Mamidala and Swamy Nanna, 2009; Savitri and Hardjo, 2019). However, only few in depth analyses of fruit development have been conducted. To our knowledge, this work is the first to report metabolite/pigment profiles of tissue culture produced MicroTom fruit (**Figures 2A, B**).

Results show that MicroTom can produce fruit *in vitro* (**Figure 2A**), which is not observed in all plants. Lycopene content of unripen fruit ranged from 1.67 – 12.49 µg g^-1^ dry weight, ripening fruit from 642.99 – 1748.41 µg g^-1^ dry weight, and fully ripe fruit from 1726.79 – 2029.67 µg g^-1^ dry weight (**Figure 2B**). Lycopene values are within reasonably similar ranges to those reported in previous tomato studies (Baranska et al., 2006; Mendelová et al., 2013; Coyago-Cruz et al., 2018; Aono et al., 2021). Total carotenoids of *in vitro* MicroTom fruit ranged from 21.72 – 54.88 µg g^-1^ dry before ripening, 1027.38 – 2637.85 µg g^-1^ dry weight during ripening, and from 2478.29 – 2918.67 µg g^-1^ dry weight when fully ripe (**Figure 2B**). These values are also in the ranges of those reported in previous tomato studies (Suzuki et al., 2015; Coyago-Cruz et al., 2018; Aono et al., 2021). Lycopene is considered the major of carotenoid produced by red tomatoes (Palmitessa et al., 2021). Photo-selective shading can increase lycopene content while reducing β-carotene of field tomatoes (Ilić et al., 2012a; Ilić et al., 2012b) Supplementation with red and blue light can also promote tomato lycopene synthesis (Palmitessa et al., 2021). The *in vitro* MicroTom produced with low intensity polychromatic LEDs appear to follow these trends (**Figures 2A, B**). Ethylene plays a central role in controlling fruit ripening and carotenoid synthesis, by which ethylene insensitivity results in fruit with low amounts of β-carotene and lycopene (Télef et al., 2006). On this note, high concentrations of ethylene can accumulate in culture vessels (Biddington, 1992), perhaps leading to increased lycopene concentrations, a factor to consider for follow-up experiments. Additionally, Télef et al. (2006) found that a reduction in exogenous sucrose delayed the accumulation of phytoene and lycopene, with no effect on β-carotene in ripening *in vitro* tomato pericarp discs (Télef et al., 2006), yet another aspect for future study. Despite *in vitro* production, fruit ripening process appears to be largely normal (**Figures 2A, B**). Since this was not a side-by-side, controlled comparison, additional experiments must be completed to tackle any differences in genetic backgrounds and analytical methods to validate the similarities observed. Ultimately, these findings represent the first accounts of *in vitro* MicroTom fruit pigments and the featured similarities to *ex vitro* tomato fruit show promising support for the presented perspective.

## Parthenocarpic fruiting of MicroTom in tissue culture

An additional observation relating to *in vitro* MicroTom fruit (**Figure 2A**) is their lack of seeds. Harvested fruits produced no seeds, bringing the fertility of these specimens into question. The flowers were not intentionally pollinated, and there was no wind movement in the vessels to promote pollination. Tomato flowers can self-pollinate, and even without wind it is likely that some pollen could have fallen on stigmas. However, even immature seeds were absent. In the future, hand-pollination could determine the viability of pollen and receptivity of stigma *in vitro*. Nevertheless, different combinations of auxin, cytokinins, and gibberellins can promote fertilization-independent fruit induction in certain plants, like tomato (Pandolfini, 2009). Auxin and gibberellins are central factors affecting parthenocarpic tomato development (Gorguet et al., 2005). It has been suggested that pollination and fertilization -related signals can be induced with exogenous applications of auxin, and up-stream initiation of auxin-induced fruiting can be facilitated with gibberellin treatments (Pandolfini, 2009). In practice, concurrent application of GA_3_ and 2,4-D to greenhouse MicroTom allows development of parthenocarpic fruit (Serrani et al., 2007). Although there were no exogenous plant growth regulators used in the current work, different environmental cues of *in vitro* systems can facilitate redistribution of internal phytohormone concentrations (Pepe et al., 2021), perhaps leading to seedless fruit development. Alternatively, ethylene impacts auxin action in promoting parthenocarpy, although a reduction in ethylene responsiveness generally results in parthenocarpic fruit (Lin et al., 2008; Martínez et al., 2013; Shinozaki et al., 2018). Thus, ethylene was likely not a principal factor here, since ethylene accumulation is a common occurrence *in vitro* (Biddington, 1992). Considering these factors, the availability of hormone-insensitive MicroTom mutants, along with the simplicity at which growth regulators can be added to tissue culture media identifies *in vitro* production of MicroTom as an ideal platform to study hormonal influences on fruit development.

## Conclusion

The presented perspective is to merge the model organism, MicroTom, with an *in vitro* system to create a powerful planform for modeling plant growth and developmental in response to highly controllable abiotic conditions, in the absence of confounding variables. As a phenotyping tool, this marriage of a model plant, in tissue culture, using gas-exchange techniques makes it possible to narrow down composite treatments before they are replicated on a larger scale using conventional systems. While further validation studies are needed, similar trends among *in vitro* and *ex vitro* MicroTom responses reported here endorse the use of tissue culture to model abiotic conditioning and stress responses. Additionally, the extensive gene bank of MicroTom mutants available gives higher value to the tissue culture system for creating more powerful genotyping and pathway mapping experiments. Since this study focuses on directly comparing *in vitro* methods to growth chamber methods, it was necessary to include sucrose as a standard tissue culture media component. Thus, these experiments must be repeated without exogenous carbohydrates, to quantify potential differences between *in vitro* and growth chamber methods. The similarities between *in vitro ex vitro* fruit and discrepancies related seed development further indicate the need for additional experiments of this nature. By improving and employing *in vitro* MicroTom techniques, it is possible to execute replicable, statistically valid, high throughput genotyping and dynamic phenotyping experiments, with major relevance for follow-up field and greenhouse studies.

## Data availability statement

The original contributions presented in the study are included in the article/**Supplementary Material**. Further inquiries can be directed to the corresponding author.

## Author contributions

MP, TRJGM, EDL, MH, AMPJ, and BG collaboratively conceptualized and designed the study. MP cultured *in vitro* plants, ran the gas exchange system, drafted, and revised the manuscript. TRJGM grew *ex vitro* plants, ran the gas exchange system, analyzed data, edited the manuscript. EDL ran the gas exchange system, analyzed data, edited the manuscript. MH cultured *in vitro* plants and edited the manuscript. NR analyzed data and edited the manuscript. AMPJ and BG overlooked the project, provided laboratory space and equipment, acquired funding, and edited the manuscript. All authors contributed to this work and approve this manuscript for submission.

## Funding

This publication was supported with grants provided by OMAFRA Alliance and OVGV (UG-T1-2021-100933) to BG and an NSERC discovery grant (RGPIN-2016-06252) awarded to AMPJ.

## Acknowledgments

OMAFRA and NSERK committees for providing funding, and University of Guelph for providing laboratory space.

## Conflict of interest

The authors declare that the research was conducted in the absence of any commercial or financial relationships that could be construed as a potential conflict of interest.

## Publisher’s note

All claims expressed in this article are solely those of the authors and do not necessarily represent those of their affiliated organizations, or those of the publisher, the editors and the reviewers. Any product that may be evaluated in this article, or claim that may be made by its manufacturer, is not guaranteed or endorsed by the publisher.

## References

[B1] Anberbir A. H. (2014). Determination of the concentration of lycopene in tomato by using absorption spectroscopy (Addis Ababa, Ethiopia).

[B2] Aono Y. Asikin Y. Wang N. Tieman D. Klee H. Kusano M. (2021). High-throughput chlorophyll and carotenoid profiling reveals positive associations with sugar and apocarotenoid volatile content in fruits of tomato varieties in modern and wild accessions. Metabolites 11, 1–12. doi: 10.3390/metabo11060398 PMC8233878 34207208

[B3] Arie T. Takahashi H. Kodama M. Teraoka T. (2007). Tomato as a model plant for plant-pathogen interactions. Plant Biotechnol. 24, 135–147. doi: 10.5511/plantbiotechnology.24.135

[B4] Baranska M. Schütze W. Schulz H. (2006). Determination of lycopene and β-carotene content in tomato fruits and related products: Comparison of FT-raman, ATR-IR, and NIR spectroscopy. Anal. Chem. 78, 8456–8461. doi: 10.1021/ac061220j 17165839

[B5] Biddington N. L. (1992). The influence of ethylene in plant tissue culture. Plant Growth Regul. 11, 173–187. doi: 10.1007/BF00024072

[B6] Bodhipadma K. Leung D. W. M. (2003). *In vitro* fruiting and seed set of capsicum annuum l. cv. sweet banana. Vitr Cell Dev. Biol. - Plant 39, 536–539. doi: 10.1079/IVP2003441

[B7] Coyago-Cruz E. Corell M. Moriana A. Hernanz D. Benítez-González A. M. Stinco C. M. . (2018). Antioxidants (carotenoids and phenolics) profile of cherry tomatoes as influenced by deficit irrigation, ripening and cluster. Food Chem. 240, 870–884. doi: 10.1016/j.foodchem.2017.08.028 28946354

[B8] Dan Y. Yan H. Munyikwa T. Dong J. Zhang Y. Armstrong C. L. (2006). MicroTom - a high-throughput model transformation system for functional genomics. Plant Cell Rep. 25, 432–441. doi: 10.1007/s00299-005-0084-3 16341726

[B9] Dutton R. G. Jiao J. Tsujita M. J. Grodzinski B. (1988). Whole plant CO_2_ exchange measurements for nondestructive estimation of growth. Plant Physiol. 86, 355–358. doi: 10.1104/pp.86.2.355 16665912 PMC1054488

[B10] Franklin G. Pius P. K. Ignacimuthu S. (2000). Factors affecting *in vitro* flowering and fruiting of green pea (Pisum sativum l.). Euphytica 115, 65–74. doi: 10.1023/A:1003982900117

[B11] Gago J. Martínez-Núñez L. Landín M. Flexas J. Gallego P. P. (2014). Modeling the effects of light and sucrose on *in vitro* propagated plants: A multiscale system analysis using artificial intelligence technology. PloS One 9, e85989. doi: 10.1371/journal.pone.0085989 24465829 PMC3896442

[B12] Gorguet B. Van Heusden A. W. Lindhout P. (2005). Parthenocarpic fruit development in tomato. Plant Biol. 7, 131–139. doi: 10.1055/s-2005-837494 15822008

[B13] Ilić Z. S. Milenković L. Stanojević L. Cvetković D. Fallik E. (2012a). Effects of the modification of light intensity by color shade nets on yield and quality of tomato fruits. Sci. Hortic. (Amsterdam) 139, 90–95. doi: 10.1016/j.scienta.2012.03.009

[B14] Ilić Z. S. Milenković L. Šunić L. Stanojević L. Bodroža-solarov M. Marinković D. (2012b). “Tomato fruits quality as affected by light intensity using color shade nets,” in 47th Croatian and 7th International Symposium on Agriculture. Opatija, Croatia (Hrvatska), 414–418.

[B15] Jiao J. Tsujita M. J. Grodzinski B. (1991). Influence of temperature on net CO 2 exchange in roses. Can. J. Plant Sci. 71, 235–243. doi: 10.4141/cjps91-033

[B16] Kozai T. Afreen F. Zobayed S. M. A. (2005). Photoautotrophic (sugar-free medium) micropropagation as a new micropropagation and transplant production system. Photoautotrophic (sugar-free medium) Micropropagat as New Micropropagat Transplant. Product System. 1, 1–316. doi: 10.1007/1-4020-3126-2

[B17] Lembrechts R. Ceusters N. De Proft M. P. Ceusters J. (2017). Sugar and starch dynamics in the medium-root-leaf system indicate possibilities to optimize plant tissue culture. Sci. Hortic. (Amsterdam). 224, 226–231. doi: 10.1016/j.scienta.2017.06.015

[B18] Leonardos E. D. Grodzinski B. (2014). “Quantifying immediate carbon export from source leaves,” in Handbook of plant and crop physiology, second edition (University of Arizona, Tucson Arizona: Marcel Dekker, Inc). doi: 10.1201/9780203908426

[B19] Leonardos E. D. Grodzinski B. (2016). “Quantifying growth non-destructively using whole-plant CO2 exchange is a powerful tool for phenotyping,” in Handbook of photosynthesis. Ed. Pessarakli M. (Boca Raton, FL, USA: CRC Press), 571–589.

[B20] Li Y. Chen Y. Zhou L. You S. Deng H. Chen Ya . (2020). MicroTom metabolic network: Rewiring tomato metabolic regulatory network throughout the growth cycle. Mol. Plant 13, 1203–1218. doi: 10.1016/j.molp.2020.06.005 32561360

[B21] Lin Z. Arciga-Reyes L. Zhong S. Alexander L. Hackett R. Wilson I. . (2008). SlTPR1, a tomato tetratricopeptide repeat protein, interacts with the ethylene receptors NR and LeETR1, modulating ethylene and auxin responses and development. J. Exp. Bot. 59, 4271–4287. doi: 10.1093/jxb/ern276 19036844 PMC2639023

[B22] Mamidala P. Swamy Nanna R. (2009). Efficient *in vitro* plant regeneration, flowering and fruiting of dwarf tomato cv. plant omi. J. South. Cross Journals. 2, 98–102.

[B23] Marie T. R. J. G. Leonardos E. D. Lanoue J. Hao X. Micallef B. J. Grodzinski B. (2022). A perspective emphasizing circadian rhythm entrainment to ensure sustainable crop production in controlled environment agriculture: Dynamic use of LED cues. Front. Sustain FoodSyst. 6. doi: 10.3389/fsufs.2022.856162

[B24] Martínez C. Manzano S. Megías Z. Garrido D. Picó B. Jamilena M. (2013). Involvement of ethylene biosynthesis and signalling in fruit set and early fruit development in zucchini squash (Cucurbita pepo l.). BMC Plant Biol. 13, 1–14. doi: 10.1186/1471-2229-13-139 PMC3856489 24053311

[B25] Mendelová A. Mendel L. Fikselová M. Czako P. (2013). Effect of drying temperature on lycopene content of processed tomatoes. Potravinarstvo 7, 141–145. doi: 10.5219/300

[B26] Monthony A. S. Page S. R. Hesami M. Jones A. M. P. (2021). The past, present and future of cannabis sativa tissue culture. Plants 10, 1–29. doi: 10.3390/plants10010185 PMC7835777 33478171

[B27] Morini S. Melai M. (2005). Net CO2 exchange rate of *in vitro* plum cultures during growth evolution at different photosynthetic photon flux density. Sci. Hortic. (Amsterdam) 105, 197–211. doi: 10.1016/j.scienta.2005.01.016

[B28] Norikane A. Takamura T. Morokuma M. Tanaka M. (2010). *In vitro* growth and single-leaf photosynthetic response of cymbidium plantlets to super-elevated CO2 under cold cathode fluorescent lamps. Plant Cell Rep. 29, 273–283. doi: 10.1007/s00299-010-0820-1 20094885

[B29] Palmitessa O. D. Durante M. Caretto S. Milano F. D’imperio M. Serio F. . (2021). Supplementary light differently influences physico-chemical parameters and antioxidant compounds of tomato fruits hybrids. Antioxidants 10, 1–15. doi: 10.3390/antiox10050687 PMC8145936 33925644

[B30] Pandolfini T. (2009). Seedless fruit production by hormonal regulation of fruit set. Nutrients 1, 168–177. doi: 10.3390/nu1020168 22253976 PMC3257607

[B31] Park E. J. Lee S. D. Chung E. J. Lee M. H. Um H. Y. Murugaiyan S. . (2007). MicroTom - a model plant system to study bacterial wilt by ralstonia solanacearum. Plant Pathol. J. 23 (4), 239–244. doi: 10.5423/PPJ.2007.23.4.239

[B32] Pepe M. Hesami M. Small F. Jones A. M. P. (2021). Comparative analysis of machine learning and evolutionary optimization algorithms for precision micropropagation of cannabis sativa: Prediction and validation of *in vitro* shoot growth and development based on the optimization of light and carbohydrate sources. Front. Plant Sci. 12. doi: 10.3389/fpls.2021.757869 PMC8566924 34745189

[B33] Pepe M. Leonardos E. D. Marie T. R. J. G. Kyne S. T. Hesami M. Jones A. M. P. . (2022). A noninvasive gas exchange method to test and model photosynthetic proficiency and growth rates of *In vitro* plant Cultures : Preliminary implication for cannabis sativa l. Biology 11, 1–14. doi: 10.3390/biology11050729 PMC9139056 35625457

[B34] Rocha P. S. G. Oliveira R. P. Scivittaro W. B. (2013). Sugarcane micropropagation using light emitting diodes and adjustment in growth-medium sucrose concentration. Ciec. Rural. 43, 1168–1173. doi: 10.1590/s0103-84782013000700005

[B35] Rybczyński J. J. Borkowska B. Fiuk A. Gawrońska H. Śliwińska E. Mikuła A. (2007). Effect of sucrose concentration on photosynthetic activity of *in vitro* cultures gentiana kurroo (Royle) germlings. Acta Physiol. Plant 29, 445–453. doi: 10.1007/s11738-007-0054-1

[B36] Savitri W. Hardjo P. H. (2019). Induction of flowering and fruiting in plantlets of tomato (Lycopersicon esculentum mill.). MPI (Media Pharm. Indones. 2, 57–63. doi: 10.24123/mpi.v2i2.1303

[B37] Serrani J. C. Fos M. Atarés A. García-Martínez J. L. (2007). Effect of gibberellin and auxin on parthenocarpic fruit growth induction in the cv micro-tom of tomato. J. Plant Growth Regul. 26, 211–221. doi: 10.1007/s00344-007-9014-7

[B38] Sheeja T. E. Mandal A. B. (2003). *In vitro* flowering and fruiting in tomato (Lycopersicon esculentum mill.). *Asia-pacific J. Mol Biol.* . Biotechnol 11, 37–42.

[B39] Shinozaki Y. Ezura H. Ariizumi T. (2018). The role of ethylene in the regulation of ovary senescence and fruit set in tomato (Solanum lycopersicum). Plant Signal Behav. 13, 1–4. doi: 10.1080/15592324.2016.1146844 PMC5933915 26934126

[B40] Song Y. Jiang C. Gao L. (2016). Polychromatic supplemental lighting from underneath canopy is more effective to enhance tomato plant development by improving leaf photosynthesis and stomatal regulation. Front. Plant Sci. 7. doi: 10.3389/fpls.2016.01832 PMC5145862 28018376

[B41] Suzuki M. Takahashi S. Kondo T. Dohra H. Ito Y. Kiriiwa Y. . (2015). Plastid proteomic analysis in tomato fruit development. PloS One 10, 1–25. doi: 10.1371/journal.pone.0137266 PMC4570674 26371478

[B42] Takahashi H. Shimizu A. Arie T. Rosmalawati S. Fukushima S. Kikuchi M. . (2005). Catalog of micro-tom tomato responses to common fungal, bacterial, and viral pathogens. J. Gen. Plant Pathol. 71, 8–22. doi: 10.1007/s10327-004-0168-x

[B43] Télef N. Stammitti-Bert L. Mortain-Bertrand A. Maucourt M. Carde J. P. Rolin D. . (2006). Sucrose deficiency delays lycopene accumulation in tomato fruit pericarp discs. Plant Mol. Biol. 62, 453–469. doi: 10.1007/s11103-006-9033-y 16915514

[B44] Tisserat B. Galletta P. D. (1995). *In vitro* flowering and fruiting of capsicum fruitescens l. HortScience 30, 130–132. doi: 10.21273/hortsci.30.1.130

[B45] Vitale E. Velikova V. Tsonev T. (2022). Manipulation of light quality is an effective tool to regulate photosynthetic capacity and fruit antioxidant properties of solanum lycopersicum l. cv. ‘ microtom ‘ in a controlled environment. PeerJ, 10, 1–23. doi: 10.7717/peerj.13677 PMC9252183 35795173

[B46] Walli M. H. Jasim H. M. Ali F. H. (2019). Effect of exchange of gases for tissue culture vessels to produce thmeristem of solanum tuberosum *in vitro.* int J drug deliv. Technol 9, 98–101. doi: 10.25258/ijddt.v9i3.27

