# Supplementary material for: Tissue culture coupled with a gas exchange system offers new perspectives on phenotyping the developmental biology of Solanum lycopersicum L. cv. ‘MicroTom’
Source: Front Plant Sci. 2022 Nov 10;13:1025477. doi: 10.3389/fpls.2022.1025477 (PMC9691339; doi:10.3389/fpls.2022.1025477)
Supplement: Supplementary file 1 [file DataSheet_1.pdf]

Materials and Methods:

Tissue Culture Protocols

100 *Solanum lycopersicum* L. cv. 'Microtom' (MicroTom) seeds were germinated under polychromatic red/blue/white LEDs at 50 µmol m^{-2} s^{-1} PPFD, at 26 ^{o}C day/night, on MS media with 2.2 g/L (w/v) Phytagel and 30 g/L (w/v) sucrose pH of 5.7 adjusted with potassium hydroxide before autoclaving. Media components were obtained from PhytoTech Labs, Inc. (Lenexa, KS, USA). Once first true leaves emerged, 23 uniform specimens were sub-cultured to grow for 2-weeks in custom made gas exchange magenta boxes described by Pepe *et al.* (2022) containing identical media and under identical conditions as those for germination. Once plants grew to appropriate sizes, the 4 most uniform plants were chosen for evaluation using the tissue culture gas exchange experimental system.

Tissue culture gas exchange experiments were conducted using the methods presented by Pepe et al. (2022) and Leonardos and Grodzinski (2014). Net carbon exchange rates (NCER) were measured using the same 4 plants a total diurnal 48-hr period. An initial 12-hr of light at the previously acclimated 50 µmol m^{-2} s^{-1} PPFD was followed by 12-hr of darkness. Light intensity was then doubled to 100 µmol m^{-2} s^{-1} PPFD for 12-hr, followed by 12-hr of darkness. During experimentation, relative humidity ranged from 85-90%. Graphs were created using the methods presented in Pepe et al. (2022).

Growth Cabinet Protocols

MicroTom seeds were germinated in darkness and grown in 1 L pots with potting soil. After germination, plants were placed under 200 µmol m^{-2} s^{-1} PPFD in growth cabinets (BioChambers Inc, MB, Canada), at 21 ^{o}C day/night, and fertigated with 20-8-20 (Plant Products Inc, ON, Canada) at an EC of 1.8-2 and adjusted to a pH of 5.8 with phosphoric acid. Four of the most uniform 8-week-old plants were selected for whole-plant gas exchange experimentation.

Whole-plant NCERs of these 4 plants were obtained over a total diurnal 48-hr period using a whole-plant gas exchange system previously described (Dutton *et al.*, 1988; Leonardos and Groddzinski, 2016). An initial 12-hr of light at the previously acclimated 200 µmol m^{-2} s^{-1} PPFD was followed by 12-hr of darkness. Light intensity was then doubled to 400 µmol m^{-2} s^{-1} PPFD for 12-hr, followed by 12-hr of darkness. Relative humidity was between 60-70%.

Gas exchange experiments were conducted on tissue culture plants during the late vegetative stage, while cabinet plants in the early flowering stage were used. In each gas exchange system, gas exchange measurements were conducted when [CO_2] was maintained at 400 ppm. NCERs during light and dark periods were integrated, generating C-gains over the entire 48-hr period.

Fruit Analysis Protocols

The fruit analyzed were not produced from the plants used in the gas exchange experiments. Analyzed fruit were collected from alternative cultures growing in identical media and under

identical conditions as specimens used for gas exchange analysis. Four subsamples for each ripening phase were harvested for analysis.

For lycopene and total carotenoid analysis, extracts were prepared by grinding samples in pure acetone using Aono et al. (2021) method. Lyophilized tissue was ground in Retsch MM301 Mixer Mill at 30 Hz for 10-minutes. 2-5 mg of ground up tissue was then homogenized in 0.5 mL of pure acetone in 2 mL tubes at 15 HZ for 10-minutes. Samples were centrifuged at 15000 g for 5-minutes at 20 °C, then the supernatant was transferred into fresh tubes. This process was repeated twice. Samples were kept on ice until analysis. Total carotenoid content was calculated using Lichtenthaler equations for pure acetone (Lichtenthaler, 1987). Lycopene content was calculated using the Beer-Lambert Law, where the molar extinction coefficient value was used from Anberbir (2014) at wavelength 505. Results were expressed as ug/g dry weight.

References:

Anberbir, A. H. (2014). Determination of the Concentration of Lycopene in Tomato By Using Absorption Spectroscopy. *Addis Ababa, Ethiopia*. 32.

Aono, Y., Asikin, Y., Wang, N., Tieman, D., Klee, H., Kusano, M. (2021). High-throughput chlorophyll and carotenoid profiling reveals positive associations with sugar and apocarotenoid volatile content in fruits of tomato varieties in modern and wild accessions. *Metabolites*, 11, 1-12. doi: 10.3390/metabo11060398

Lichtenthaler, H.K. (1987). Chlorophylls and Carotenoids: Pigments of Photosynthetic Biomembranes. *Methods Enzymol*. 148, 350–382. doi: 10.1016/0076-6879(87)48036-1

Pepe, M., Leonardos, E.D., Marie, T.R.J.G., Kyne, S.T., Hesami, M., Jones, A.M.P., Grodzinski, B. (2022). A Noninvasive Gas Exchange Method to Test and Model Photosynthetic Proficiency and Growth Rates of In Vitro Plant Cultures : Preliminary Implication for Cannabis sativa L. *Biology*. 11, 1–14. doi: 10.3390/biology11050729
